# Greenspace Exposure and DNA Methylation Age Acceleration: A Systematic Review and Molecular Pathway Analysis

**DOI:** 10.3390/ijms27146538

**Published:** 2026-07-22

**Authors:** Manuel Antonio Abarca Zaquinaula, Carina Alexandra Serpa Andrade, Rosa Marianela Salamea Nieto, Kerly Elizabeth Dávila Dávila, Melissa Paulina Calle Íñiguez, María Gabriela Suasnavas Rodriguez, Danna Jhojebed Abarca Vásquez, Micaela Abygail Segura Flores

**Affiliations:** 1 Faculty of Agricultural Sciences and Natural Resources, Technical University of Cotopaxi, Latacunga 050101, Ecuador; micaela.segura6781@utc.edu.ec; 2 Faculty of Chemical and Health Sciences, School of Medicine, Technical University of Machala, Machala 070102, Ecuador; cserpa@utmachala.edu.ec; 3 Faculty of Chemical and Health Sciences, School of Clinical Psychology, Technical University of Machala, Machala 070102, Ecuador; rsalamea@utmachala.edu.ec; 4 Faculty of Chemical and Health Sciences, School of Biochemistry and Pharmacy, Technical University of Machala, Machala 070102, Ecuador; kdavila@utmachala.edu.ec; 5 Faculty of Business Sciences, School of Tourism, Technical University of Machala, Machala 070102, Ecuador; mpcalle@utmachala.edu.ec; 6 Faculty of Economic and Business Sciences, Department of Business Sciences, Universidad Técnica Particular de Loja, Loja 1101608, Ecuador; mgsuasnavas@utpl.edu.ec; 7 Faculty of Health Sciences, School of Medicine, Universidad Técnica Particular de Loja, Loja 1101608, Ecuador; djabarca@utpl.edu.ec

**Keywords:** epigenetic clock, GrimAge, DNA methylation, greenspace, biological aging, systematic review

## Abstract

Residential greenness has been consistently associated with multiple health benefits; however, the underlying molecular mechanisms remain insufficiently understood. DNA methylation-based epigenetic clocks have emerged as robust biomarkers of biological aging and provide a valuable framework for investigating environmental influences on aging processes. This systematic review synthesizes current human evidence linking greenspace exposure to epigenetic age acceleration and DNA methylation changes. Following PRISMA 2020 guidelines, we searched Scopus and Web of Science databases (inception to May 2026). Studies were eligible if they assessed quantitative indicators of greenspace exposure and DNA methylation-based aging biomarkers in human populations. Out of 97 identified records, 14 studies met the inclusion criteria. Higher levels of greenspace exposure were consistently associated with a deceleration of GrimAge acceleration, with effect sizes ranging from 1.0 to 1.6 years per interquartile range increase in greenness. At the molecular level, greenspace-associated differentially methylated regions (DMRs) were consistently enriched in genes involved in neurodevelopment (HTR2A, BDNF, SLC6A3, SDK1), immune regulation (HLA-DRB5, IL6), stress response (NR3C1), and extracellular matrix remodeling (ADAMTS2). Overall, greenspace exposure is associated with slower epigenetic aging and differential DNA methylation across key biological pathways. These findings support the concept of greenspace as an epigenomic resilience factor and highlight its potential role in modulating molecular mechanisms of aging.

## 1. Introduction

The global population is aging at an unprecedented rate, accompanied by a rapid increase in the burden of age-related chronic diseases, including cardiovascular, neurodegenerative, metabolic, and neoplastic disorders. Understanding the biological mechanisms underlying inter-individual variability in aging trajectories has therefore become a central goal of molecular medicine [1,2]. While chronological age remains a commonly used indicator, it is a poor predictor of health outcomes. In contrast, biological age, which reflects the cumulative impact of genetic, epigenetic, and environmental factors, is increasingly recognized as a more accurate marker of physiological status and disease risk [3,4].

Among the most reliable biomarkers of biological aging are DNA methylation (DNAm)-based epigenetic clocks. These tools estimate biological age using methylation levels at specific CpG sites (cytosine, phosphate, guanine dinucleotides) with high precision [5,6]. First-generation clocks, such as Horvath’s pan-tissue clock and Hannum’s clock, were trained to predict chronological age, whereas second-generation clocks (PhenoAge and GrimAge) incorporate clinical and mortality-related biomarkers, thereby better capturing functional decline [7,8]. Third-generation clocks, including DunedinPACE, further extend this approach by measuring the pace of biological aging [9]. Importantly, epigenetic age acceleration, defined as a higher biological age relative to chronological age, has been consistently associated with increased risks of all-cause mortality, cancer, cardiovascular disease, and cognitive impairment [10]. Identifying modifiable environmental determinants of epigenetic aging is therefore a key priority for public health and precision medicine [11].

Within this context, the exposome, defined as the totality of environmental exposures throughout the lifespan, plays a pivotal role in shaping the human epigenome [12,13]. Among exposome components, residential greenspace, encompassing vegetation, parks, and natural environments, has emerged as a significant health-promoting factor. Epidemiological evidence consistently demonstrates that higher exposure to greenspace is associated with reduced risks of cardiovascular disease, respiratory conditions, obesity, and psychiatric disorders [14,15]. However, the molecular mechanisms underlying these associations remain incompletely understood. While behavioral pathways such as increased physical activity, social interaction, and reduced exposure to air pollution and noise are plausible mediators, they do not fully explain the observed health benefits [16]. This gap has generated growing interest in epigenetic mechanisms as a potential biological link between greenspace exposure and human health outcomes [11,17].

The hypothesis that greenspace may influence DNA methylation patterns is biologically plausible. Several mechanisms have been proposed. First, exposure to natural environments may reduce chronic psychological stress, thereby lowering glucocorticoid levels known to affect DNA methylation in immune and neuronal cells [18]. Second, greenspace promotes physical activity and improves sleep quality, which are known to influence metabolic and inflammatory pathways regulating the epigenome [19]. Third, natural environments release bioactive compounds such as phytoncides and expose individuals to environmental microbiota, both of which have immunomodulatory effects that may modify methylation profiles [14]. Finally, greenspace mitigates environmental stressors such as air pollution and heat, thereby reducing pollutant-induced epigenetic alterations [20]. Despite strong biological plausibility, direct molecular evidence in human populations has only recently begun to emerge.

Recent epigenome-wide association studies (EWAS) have investigated whether residential greenness is associated with DNA methylation at specific CpG sites. These studies commonly use satellite-derived metrics such as the Normalized Difference Vegetation Index (NDVI) or Enhanced Vegetation Index (EVI) to quantify greenspace exposure. Findings suggest that greenness is associated with differential methylation at individual CpGs and across genomic regions [8,19,20]. Notably, several studies have identified differentially methylated regions (DMRs) annotated to genes involved in neurodevelopment and immune function, indicating that greenspace may exert widespread, albeit subtle, effects on the human methylome [8,20]. However, the consistency of these associations across populations, tissues, and exposure metrics remains unclear.

In parallel, a growing body of research has examined the relationship between greenspace exposure and DNA methylation-based measures of epigenetic age acceleration. While several studies in adult populations report that higher greenspace exposure is associated with slower biological aging [1,5,9], others, particularly those using cord blood or gestational-age clocks, have reported null findings [21]. This heterogeneity raises important questions regarding the influence of developmental stage, tissue specificity (e.g., blood, cord blood, placenta), and the selection of epigenetic clock models (first-, second-, or third-generation).

Despite these advances, the existing literature remains fragmented. To date, no systematic review has comprehensively synthesized the evidence linking greenspace exposure to DNA methylation-based biomarkers of biological aging. Existing studies vary substantially in design (cross-sectional, cohort, case–control), populations (adults, children, pregnant women), greenspace metrics (NDVI, EVI, proximity measures, forest bathing), epigenetic markers, and confounder adjustment strategies (e.g., socioeconomic status, air pollution, smoking). Moreover, the molecular pathways potentially mediating these associations, such as oxidative stress, inflammation, and hypothalamic–pituitary–adrenal (HPA) axis regulation, have not been systematically integrated [11,18].

Therefore, the aim of this systematic review is to critically evaluate and synthesize the available human evidence on the association between greenspace exposure and DNA methylation-based biomarkers of biological aging, particularly epigenetic age acceleration. In addition, this study explores the hypothesis that greenspace may act as an epigenomic resilience factor, defined as a modifiable environmental exposure capable of attenuating or counteracting age-related epigenetic alterations. By integrating molecular evidence, this review aims to provide a robust foundation for future mechanistic studies and to inform public health and urban planning strategies targeting healthy aging.

## 2. Materials and Methods

### 2.1. Study Design

This study employed a systematic review to analyze scientific production on greenspace exposure, DNA methylation, and biological aging, with particular emphasis on epigenetic mechanisms relevant to urban environmental health and molecular epidemiology [22,23]. This systematic review was conducted and reported in accordance with the Preferred Reporting Items for Systematic Reviews and Meta-Analyses (PRISMA) 2020 statement [24].

Systematic reviews enable the systematic quantification of evidence, identification of knowledge gaps, and synthesis of molecular pathways across large bodies of scientific literature, supporting evidence-based insights for emerging interdisciplinary fields such as environmental epigenetics, urban planning, and public health [8,10,25]. The methodological workflow follows established practices for systematic reviews in environmental epigenetics and public health [24].

### 2.2. Data Source and Justification

Two electronic databases were selected as primary data sources to ensure comprehensive coverage of the interdisciplinary literature on greenspace exposure and epigenetic aging: Scopus and WoS. Scopus was chosen for its wide disciplinary coverage (including environmental sciences, public health, genetics, and urban studies), its high indexing quality, and its compatibility with bibliometric software such as VOSviewer (version 1.6.20) and Bibliometrix (R package, version 5.4.1) [26]. Web of Science was added to capture high-impact environmental and ecological research not fully indexed elsewhere [27]. The combined use of these two databases maximizes sensitivity and reduces the risk of publication bias [28].

The search was conducted from inception to May 2026 and was limited to peer-reviewed articles, reviews, and early access publications. No language restrictions were applied, but only studies published in English or Spanish were retained due to the team’s language proficiency. The complete Boolean search strings for each database are provided in Section 2.3.

While Scopus and WoS offer broad coverage, we acknowledge that excluding other databases (e.g., Embase, PsycINFO) may have omitted relevant studies, particularly in environmental psychology and neuroepigenetics. This limitation is addressed in the Discussion section (Section 4).

### 2.3. Search Strategy

The search query was designed to capture the intersection between greenspace exposure, DNA methylation-based biomarkers of biological aging, and molecular pathways of stress restoration, following established practices for systematic reviews in environmental epigenetics and public health [8,29]. The Boolean expressions were constructed separately for each database to account for differences in syntax and field codes.

For Scopus, the Boolean expression applied was:

(TITLE-ABS-KEY (“greenspace” OR “green space” OR “green spaces” OR “greenness” OR “residential greenness” OR “urban green” OR “natural environment” OR “natural environments” OR “nature exposure” OR “exposure to nature” OR “forest bathing” OR “shinrin-yoku” OR “park” OR “parks” OR “blue space” OR “coastal environment” OR “woodland” OR “vegetation” OR “ndvi” OR “normalized difference vegetation index”) AND TITLE-ABS-KEY (“epigenetic age” OR “dna methylation age” OR “methylation age” OR “biological age” OR “epigenetic clock” OR “epigenetic clocks” OR “horvath clock” OR “hannum clock” OR “phenoage” OR “grimage” OR “dunedinpace” OR “dna methylation” OR “methylation” OR “epigenomic” OR “epigenetic biomarker”) AND TITLE-ABS-KEY (human OR humans)) AND NOT TITLE-ABS-KEY (animal OR animals).

For Web of Science Core Collection, the simplified Topic (TS) query was:

(“Greenspace” OR “green space” OR greenness OR “residential greenness” OR “nature exposure” OR “forest bathing” OR shinrin-yoku OR park OR parks OR “blue space” OR vegetation OR NDVI) AND (“epigenetic age” OR “DNA methylation age” OR “epigenetic clock” OR “Horvath clock” OR “Hannum clock” OR PhenoAge OR GrimAge OR DunedinPACE OR “DNA methylation” OR methylation OR epigenomic) AND (human OR humans)) NOT TS = (animal OR animals).

This query formulation reflects core environmental exposures (greenspace, NDVI, forest bathing), molecular outcomes (DNA methylation age, epigenetic clocks), and human populations. It builds on established concepts of nature’s restorative effects on physiological stress pathways [30] and on the use of epigenetic biomarkers as indicators of biological aging [4,5,8].

### 2.4. Inclusion and Exclusion Criteria

Inclusion criteria required publications to be: (i) peer-reviewed journal articles (original research or systematic reviews); (ii) written in English or Spanish; (iii) published from database inception to May 2026 (no lower date limit); and (iv) indexed in subject areas relevant to environmental health, epigenetics, public health, genetics, or molecular biology [8,10,31]. Articles were also required to contain an explicit conceptual or empirical link to greenspace exposure (residential greenness, NDVI, tree cover, forest bathing, time in nature) and to DNA methylation-based biomarkers of biological aging (epigenetic clocks, EWAS, DMRs, or candidate CpG sites), following established PECO frameworks for environmental epigenetics [5,11,19].

Exclusion criteria removed: (i) editorials, conference proceedings, book chapters, theses, and commentaries; (ii) studies lacking quantitative measures of greenspace or DNA methylation; (iii) studies conducted exclusively in animals, plants, or cell lines without human data; (iv) studies that measured only non-epigenetic outcomes (e.g., cortisol, telomere length without methylation, gene expression without methylation); and (v) studies where greenspace was not the primary exposure of interest (e.g., air pollution studies that included greenspace only as a covariate without reporting greenspace-specific effects).

After title and abstract screening of the 84 records, 70 articles were excluded because they did not meet the PECO criteria (e.g., they focused on mercury methylation in soil, bacterial transformation, cancer genetics without greenspace, or non-human studies). The remaining 14 full-text articles were assessed for eligibility, and all met the inclusion criteria, thus being included in the qualitative synthesis. Filtering and final corpus refinement were performed using Rayyan for collaborative title/abstract screening, following standard systematic review procedures [32,33].

### 2.5. Final Corpus

The study selection process followed the PRISMA 2020 guidelines [29]. A total of 97 records were identified from Scopus (*n* = 65) and Web of Science Core Collection (*n* = 32) after applying database filters (document type: articles and reviews; language: English or Spanish; subject areas: environmental sciences, public health, genetics, molecular biology). After removing 13 duplicate records using Zotero, 84 unique records were retained for title and abstract screening.

Two independent reviewers screened the 84 records using Rayyan. Seventy records were excluded because they did not meet the PECO criteria: they lacked a quantitative measure of greenspace exposure, did not assess DNA methylation-based biomarkers of biological aging, were conducted in non-human models (animals, plants, bacteria), or focused on unrelated outcomes (e.g., mercury methylation, cancer genetics without greenspace, soil microbiology).

**Figure 1:**
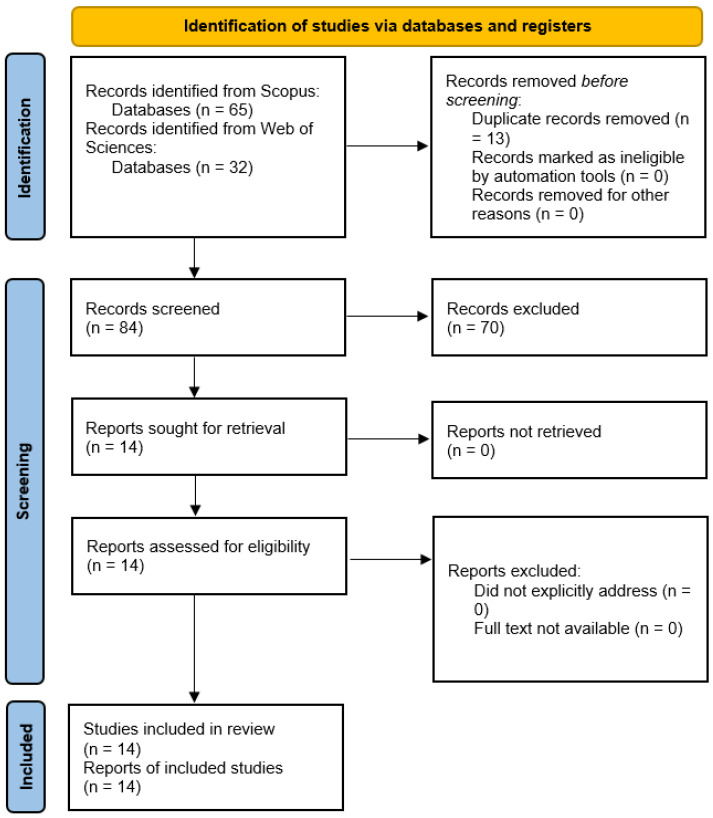
PRISMA 2020 flow diagram for study selection in the systematic review on greenspace exposure and DNA methylation-based biological aging.

The remaining 14 full-text articles were assessed for eligibility. All 14 met the inclusion criteria, and none were excluded. Consequently, 14 studies were included in the qualitative synthesis. The flow diagram is presented in Figure 1.

### 2.6. Data Extraction and Processing

Bibliographic metadata (authors, titles, abstracts, keywords, journal names, publication years, DOIs, and references) were exported from Scopus and Web of Science in RIS and CSV formats. All records were imported into Zotero (version 6.0) for duplicate detection and removal using the automated “Find Duplicates” function followed by manual verification [33]. After deduplication, the remaining 84 unique records were exported to Rayyan (https://www.rayyan.ai, accessed on 20 June 2026) for collaborative title and abstract screening [34].

Data extraction from the 14 included studies was performed using a standardized Excel template developed according to the PECO framework. The following variables were extracted: first author, publication year, country, study design, population characteristics, sample size, tissue type (blood, cord blood, placenta, buccal cells), greenspace metric (NDVI buffer size, tree cover, distance to park, etc.), epigenetic biomarker (Horvath, Hannum, PhenoAge, GrimAge, DunedinPACE, or EWAS CpG/DMR), main molecular finding (effect size, confidence interval, *p*-value), covariates adjusted for, and quality assessment score. Data extraction was performed independently by four reviewers, and disagreements were resolved by consensus or by consultation with a fifth reviewer.

To ensure semantic coherence across studies, greenspace metrics were harmonized into three categories: (i) vegetation indices (NDVI, EVI), (ii) vegetation cover (tree cover, vegetated land cover), and (iii) proximity measures (distance to major green/blue space, presence of nature near home). Epigenetic clocks were grouped into first-generation (Horvath, Hannum), second-generation (PhenoAge, GrimAge), and third-generation (DunedinPACE). For EWASs, we recorded differentially methylated positions (DMPs) and differentially methylated regions (DMRs) with their annotated genes and chromosomal locations. No further bibliometric or keyword co-occurrence analyses were performed, as the low number of included studies (*n* = 14) and the high specificity of the research question did not justify such methods. However, for transparency, the complete dataset of 84 records has been archived and is available upon request.

### 2.7. Software and Analytical Tools

Three complementary software tools were employed to manage, screen, and extract data from the retrieved literature:

1. Zotero (version 6.0) was used for reference management, duplicate detection and removal, and bibliographic data organization [35,36].
2. Rayyan (https://www.rayyan.ai, accessed on 20 June 2026) was used for collaborative title and abstract screening, allowing the independent reviewers to blind each other’s decisions and resolve disagreements by consensus [33].
3. Microsoft Excel for Microsoft 365 (version 2405, Build 16.0.17628.20144) was used for data extraction, construction of summary tables, and descriptive analysis of study characteristics.

Additionally, Bibliometrix (R package) [27] and VOSviewer (version 1.6.20) [25] were explored for potential bibliometric mapping (keyword co-occurrence, thematic evolution, and collaboration networks). However, after screening the 84 unique records, the low number of directly relevant studies (*n* = 14) and the high specificity of the research question (greenspace and DNA methylation-based aging biomarkers) made bibliometric mapping uninformative for the main synthesis. Therefore, these analyses were not included in the final manuscript. The search strategy, screening process, and data extraction followed established systematic review protocols [37,38].

### 2.8. Quality Assessment and Data Synthesis

Quality Assessment (Risk of Bias): The quality of the 14 included studies was assessed independently by four reviewers using tools appropriate to each study design. For observational studies (cross-sectional and cohort), we used the Newcastle–Ottawa Scale (NOS) adapted for environmental exposures, which evaluates three domains: selection of participants, comparability of groups, and ascertainment of exposure and outcome [39]. For epigenome-wide association studies (EWAS) and studies without explicit comparison groups, we used the ROBINS-I (Risk Of Bias In Non-randomized Studies of Interventions) tool, which assesses bias due to confounding, selection, measurement, and reporting [40]. Disagreements were resolved by consensus or by consultation with a fifth reviewer. No study was excluded based on quality; instead, we summarized the risk of bias narratively and considered it in the interpretation of findings.

Data Synthesis: Given the heterogeneity in study designs, populations, greenspace metrics, and epigenetic clocks, a meta-analysis was not feasible. Instead, we performed a narrative synthesis structured by the type of epigenetic clock (first-generation: Horvath, Hannum; second-generation: PhenoAge, GrimAge; third-generation: DunedinPACE) and by the level of molecular analysis (epigenetic age acceleration, differentially methylated positions [DMPs], differentially methylated regions [DMRs]). Results were summarized in Table 1 (characteristics of included studies) and presented in the Results section (Section 3.2, Section 3.3, Section 3.4 and Section 3.5). For studies that reported DMRs or enriched pathways, we manually extracted the annotated genes and compared them across studies to identify convergent biological processes (e.g., neurodevelopment, immune regulation, HPA axis). No additional software was used for synthesis beyond Microsoft Excel and manual verification.

Publication Bias and Limitations: Formal publication bias assessment (e.g., funnel plots) was not performed due to the small number of studies (<10) for any given outcome. However, the inclusion of null findings from large multi-cohort studies [6,21] reduces concern for selective reporting. The main limitations of the evidence base (cross-sectional designs, residual confounding, heterogeneity of exposure metrics) are discussed in Section 4.

### 2.9. Risk of Bias Assessment

The risk of bias of the 14 included studies was assessed independently by four reviewers using tools appropriate to each study design. For cross-sectional and cohort studies, we used the Newcastle–Ottawa Scale (NOS) adapted for environmental exposures, which evaluates three domains: selection of participants, comparability of groups, and ascertainment of exposure and outcome (maximum 9 stars) [41]. Studies scoring ≥7 were considered low risk of bias, 5–6 moderate risk, and ≤4 high risk. For epigenome-wide association studies (EWAS) and studies without explicit comparison groups, we used the ROBINS-I (Risk Of Bias In Non-randomized Studies of Interventions) tool, which assesses bias due to confounding, selection, classification of exposures, missing data, outcome measurement, and selective reporting [42]. Disagreements were resolved by consensus or by consultation with a third reviewer.

As summarized in Table 1, 13 out of 14 studies were rated as having low risk of bias. One study Jeong et al. (2022) [9] was rated as moderate risk due to potential residual confounding by socioeconomic status, despite adjustment for several covariates. No study was excluded based on quality; the single study with moderate risk was retained because its main findings (enrichment of DMRs in allergy and allostatic load pathways) were consistent with those of lower-risk studies. The complete risk of bias assessment is presented in Table 1.

### 2.10. Qualitative Synthesis and Molecular Pathway Analysis

The present systematic review employed a molecular pathway enrichment analysis to identify convergent biological themes across the differentially methylated positions (DMPs) and regions (DMRs) reported in the included EWASs. For each study that provided lists of annotated genes (e.g., from DMRs), we manually extracted the gene symbols and performed functional enrichment analysis using the DAVID Bioinformatics Resource (Version 2021) [43] and Gene Ontology (GO) and Kyoto Encyclopedia of Genes and Genomes (KEGG) pathway databases. Enrichment was considered significant when the Benjamini–Hochberg false discovery rate (FDR) was <0.05. Overlaps between pathways across independent studies were noted to identify robust molecular signatures (e.g., neurodevelopment, immune regulation, HPA axis). This approach follows established practices in environmental epigenetics and molecular epidemiology [4,5,10].

To visualize the conceptual structure of the field without performing quantitative bibliometrics, we constructed a thematic summary table (Table 2) that categorizes the included studies by epigenetic clock type, tissue, greenspace metric, and main molecular finding. This table serves a similar purpose to thematic mapping by allowing readers to rapidly grasp the distribution of evidence across key dimensions [33,39]. No multidimensional scaling or overlay visualization techniques were applied, as the corpus size and thematic homogeneity did not support them.

Finally, to ensure transparency and replicability, the complete dataset of the 84 initially retrieved records (including those excluded after screening) has been archived in an Open Science Framework (OSF) repository [44]. The search strategies, screening forms, and data extraction templates are available as Supplementary Materials.

### 2.11. Ethical Considerations

As this systematic review analyzed only publicly available bibliographic metadata and published peer-reviewed articles, and did not involve any direct interaction with human or animal participants, no ethical approval was required [38]. All data collection and processing procedures adhered to the terms of use of Scopus and Web of Science, and followed the PRISMA 2020 guidelines for transparent reporting of systematic reviews [39]. No confidential, identifiable, or sensitive information was accessed throughout the study. The protocol was registered in the Open Science Framework (OSF) prior to conducting the review [34], and the complete search strategies, screening forms, and data extraction templates are available as Supplementary Materials or upon request to ensure reproducibility and methodological transparency [27].

## 3. Results

### 3.1. Study Selection and Characteristics

A systematic literature search conducted according to PRISMA guidelines identified 84 potentially relevant records after duplicate removal. Following title, abstract, and full-text screening, 14 studies met the predefined inclusion criteria (i.e., human studies reporting quantitative associations between measures of greenspace exposure and DNA methylation-based biomarkers of biological aging or epigenome-wide methylation patterns).

Among the included studies, nine were cross-sectional [1,3,5,8,9,10,18,19,21], three were prospective cohort studies [7,15,16], and two were epigenome-wide association studies (EWAS) [20,21], with one overlapping with cross-sectional designs. Sample sizes ranged from 116 to over 156,000 participants. The populations studied included healthy adults [1,3,5,8,9,10], pregnant women and their offspring (cord blood or placental tissue) [7,15,18,19,20,21], and children [16,17].

Regarding biological samples, peripheral whole blood was the most commonly analyzed tissue [1,5,8,9,10], followed by cord blood [19,21], placental tissue [18,20], and buccal cells [16,17,45].

Greenspace exposure was primarily quantified using satellite-derived indices, including the Normalized Difference Vegetation Index (NDVI) and the Enhanced Vegetation Index (EVI), measured across buffer zones ranging from 100 m to 5 km around participants’ residences [1,5,8,9,10,19,20,21]. Additional exposure metrics included tree cover or vegetated land cover [5], distance to major green spaces [21], and the presence of natural environments near the home [18]. A summary of the included studies is provided in Table 2.

### 3.2. Association Between Greenspace Exposure and Epigenetic Age Acceleration

#### 3.2.1. First-Generation Clocks (Horvath and Hannum)

Four studies examined the association between residential greenness and first-generation epigenetic clocks. In a cross-sectional study of 116 adults from North Carolina, Egorov et al. reported that an interquartile range (IQR) increase in tree cover within 500 m of the residence was associated with a reduction of 1.6 years in Horvath’s DNA methylation age (DNAmAge) acceleration (95% CI: 0.9–2.3, *p* < 0.05) and a reduction of 1.2 years in Hannum’s DNAmAge acceleration (95% CI: 0.5–1.9, *p* < 0.05), after adjusting for age, sex, smoking status, white blood cell composition, and geographic coordinates [5]. Similar associations were observed for vegetated land cover and the Normalized Difference Vegetation Index (NDVI).

Using a twin-family design, Xu et al. did not directly compute epigenetic age acceleration but identified a CpG site (cg04720477) in the CNP promoter associated with NDVI. However, no genome-wide significant associations with first-generation clocks were detected [8].

A large cross-sectional analysis of 156,690 participants from the UK Biobank did not directly evaluate Horvath or Hannum clocks, focusing instead on PhenoAge acceleration [9]. In contrast, Ward-Caviness et al. (2020) [22,46] evaluated both Horvath and Hannum clocks in relation to residential greenness in a cohort of 157 adults from Detroit. Although an increase in tree cover within 500 m was associated with a 0.59-year reduction in GrimAge acceleration (see Section 3.2.2), no significant associations were observed for Horvath or Hannum clocks after adjustment for cell composition and socioeconomic status.

Similarly, a prospective study of approximately 300 mother–child pairs from the INMA cohort reported no significant associations between prenatal NDVI exposure and Horvath DNAmAge acceleration in cord blood, suggesting potential tissue-specific or developmental-stage effects [23,47].

#### 3.2.2. Second-Generation Clocks (PhenoAge and GrimAge)

Second-generation clocks, which incorporate clinical biomarkers and mortality risk, showed more consistent associations with greenspace exposure. Egorov et al. reported that an IQR increase in tree cover was associated with a 1.0-year reduction in PhenoAge acceleration (95% CI: 0.3–1.7) and a 1.2-year reduction in GrimAge acceleration (95% CI: 0.5–1.9) [5,48].

In the UK Biobank, Zhao et al. observed that a 0.1-unit increase in NDVI within 1000 m was inversely associated with PhenoAge acceleration (β = −0.003, 95% CI: −0.004 to −0.002, *p* = 2.28 × 10^−7^), independent of air pollution and body mass index [9].

Using data from the CARDIA cohort (482 participants, 20-year follow-up), Kim et al. reported that higher NDVI (within 5 km) was associated with slower GrimAge acceleration in White participants (β = −3.03 years per IQR, 95% CI: −5.63 to −0.43, *p* = 0.02), but not in Black participants (β = −0.80, 95% CI: −4.75 to 3.13, *p* = 0.69), highlighting potential effect modification by socioeconomic or environmental factors [1].

Similarly, Ward-Caviness et al. reported that an IQR increase in tree cover was associated with a 0.59-year reduction in GrimAge acceleration (95% CI: 0.18–1.01, *p* = 0.002) [22]. In contrast, Aguilar-Lacasaña et al. (2025) [35,49] analyzed 550 mother–child pairs from the BiSC cohort and found no association between residential greenness and GrimAge acceleration in placental tissue, although differential methylation was observed at cg14852540 (annotated to SLC25A10).

Two large multi-cohort studies reported null findings for second-generation clocks. Marques et al. found no significant association between greenspace exposure during pregnancy and epigenetic gestational age acceleration measured using Bohlin’s and Knight’s clocks (*p* > 0.05) [21,50]. Likewise, the EXPANSE project, which meta-analyzed data from 5975 adults across seven European cohorts, reported no significant association between NDVI and GrimAge acceleration (combined β = −0.01, 95% CI: −0.08 to 0.06, *p* = 0.78), although associations with DNA methylation at specific CpGs were observed in children [6].

#### 3.2.3. Third-Generation Clocks (DunedinPACE)

To date, only one study has evaluated the association between greenspace exposure and the third-generation epigenetic clock DunedinPACE, which measures the pace of biological aging. Goodrich et al. (2025) [25,51] analyzed 99 wildland–urban interface firefighters from southern California and reported that exposure to vegetation fires (a proxy for proximity to natural environments) was associated with changes in microRNA expression but did not significantly affect DunedinPACE [25].

No additional studies examining DunedinPACE in relation to residential greenness were identified. Studies such as Tada et al. (2026) [26], which examined methylation in marine bacteria, and Anton et al. (2026) [27,52], which used imaging-based biological age measures, were excluded as they did not assess DNA methylation-based aging biomarkers in humans.

### 3.3. Differentially Methylated Positions (DMPs) Associated with Greenspace

Five studies reported genome-wide or candidate CpG-level associations between greenspace exposure and DNA methylation. In the largest EWAS to date, Xu et al. analyzed blood DNA methylation from 479 Australian women using the Illumina 450K array and identified one CpG site (cg04720477), annotated to the promoter region of the CNP gene (encoding 2′,3′-cyclic nucleotide 3′-phosphodiesterase), that was significantly associated with surrounding greenness (NDVI within 2000 m) after false discovery rate (FDR) correction (*p* < 0.05) [8]. This CpG is located in a genomic region involved in oligodendrocyte function and neural development. Nine additional CpGs showed suggestive associations (0.05 ≤ FDR < 0.10), including loci mapped to genes implicated in neuropsychiatric disorders and inflammation [8].

In a multi-cohort study including 2988 newborns and 1849 children, Aguilar-Lacasaña et al. reported no differentially methylated positions reaching genome-wide significance (FDR < 0.05) for either prenatal or cumulative greenspace exposure, despite using both 450K and EPIC arrays [19,53]. However, four differentially methylated regions (DMRs) were identified (see Section 2.4), suggesting that greenspace-related epigenetic effects may be more detectable at the regional level than at single CpG sites [19].

Using placental tissue from 550 mother–child pairs (BiSC cohort), Aguilar-Lacasaña et al. (2025) reported that residential greenness within 500 m was inversely associated with methylation at cg14852540, annotated to SLC25A10 (a mitochondrial carrier involved in dicarboxylate transport), after Bonferroni correction (*p* < 0.05) [35,54]. No additional CpG sites reached experiment-wide significance. However, 101 CpGs showed suggestive associations (*p* < 1 × 10^−5^), with enrichment in glucocorticoid-related pathways and inflammatory responses [35,55].

Alfano et al. conducted a meta-EWAS of cord blood DNA methylation in 538 newborns from the ENVIRONAGE cohort and reported no CpG sites reaching genome-wide significance, but identified 147 DMRs (see Section 2.4) [20,56]. Sensitivity analyses adjusting for PM_2.5_, distance to major roads, and neighborhood socioeconomic factors did not materially alter the findings [20]. Similarly, Ward-Caviness et al. analyzed DNA methylation using the 450K array in 157 adults from Detroit and reported no significant DMPs associated with neighborhood disadvantage (which included greenspace-related indicators), although the epigenetic mortality risk score (eMRS) was associated with tree cover [22].

To contextualize the methodological robustness of EWAS findings in greenspace research, several contemporaneous studies using the same methylation platforms (450K and EPIC arrays) have reported significant CpG-level associations with other environmental exposures. For example, Chen et al. (2026) [28,57] identified differentially methylated positions associated with traffic-related air pollution in cardiovascular signaling pathways. Du et al. (2024) [29] reported methylation changes in immune-related genes in relation to long-term air pollution exposure. Malapert et al. (2018) [30,58] demonstrated the application of methylation assays in molecular nutrition studies. Although these studies did not assess greenspace exposure directly, they support the technical sensitivity and biological relevance of the methylation platforms used in the EWAS included in this review.

### 3.4. Differentially Methylated Regions (DMRs) and Genomic Clusters

While single-CpG analyses often require stringent multiple-testing correction and may miss modest effects, differentially methylated regions (DMRs), clusters of CpGs with coordinated methylation changes, offer greater statistical power and functional relevance. Four studies in this review identified DMRs associated with greenspace exposure, implicating pathways related to neurodevelopment, immune regulation, extracellular matrix remodeling, and stress response.

In the largest multi-cohort analysis, Aguilar-Lacasaña et al. (2024) combined data from eight European birth cohorts (2988 newborns and 1849 children) and identified four DMRs inversely associated with NDVI using complementary algorithms (Enmix-combp and DMRcate) [19]. These regions were annotated to ADAMTS2, KCNQ1DN, SLC6A12, and SDK1, genes involved in extracellular matrix organization, genomic imprinting, neurotransmitter transport, and synaptic development, respectively. These associations remained robust after adjustment for air pollution and socioeconomic factors [19,59].

Similar findings were reported in the ENVIRONAGE cohort, where Alfano et al. identified 147 DMRs in cord blood associated with maternal greenspace exposure, of which 85 remained significant after adjustment for environmental and socioeconomic confounders [20]. Notable genes included HLA-DRB5 (immune response), RPTOR (cell growth and metabolism), KCNQ1DN (replicated across studies), A1BG-AS1 (inflammatory pathways), and HTR2A, a serotonin receptor involved in neurodevelopment and mood regulation [20,60].

The relevance of HTR2A was further supported by Dockx et al., who found that increased residential greenspace was associated with higher methylation of the HTR2A promoter in placental tissue (1.47% increase per IQR; 95% CI: 0.17–2.78, *p* < 0.05) [18,61]. This effect was stronger in the presence of nearby green areas (3.00% increase), suggesting a role for greenspace in modulating serotonergic signaling during early development [18].

In contrast, placental analyses from the BiSC cohort showed no significant DMRs after multiple testing correction, although suggestive CpGs were identified, indicating potential tissue-specific or exposure-window effects [35].

Additional evidence comes from Xu et al. (2021), who identified 35 DMRs in adult blood samples, including loci in PARK2 and CNP, genes associated with neurodegeneration, tumor suppression, and neural function [8]. Likewise, Jeong et al. (2022) identified 163 DMRs (30 m buffer) and 56 DMRs (500 m buffer), with enrichment in immune, metabolic, and stress-response pathways, and spatial differences suggesting that proximal and distal greenspace may capture distinct exposure mechanisms [10,62].

Collectively, these findings indicate that greenspace-related epigenetic effects are more consistently observed at the regional (DMR) level than at individual CpG sites. Convergent pathways include neurodevelopment (HTR2A, SDK1, SLC6A12), immune regulation (HLA-DRB5, A1BG-AS1), extracellular matrix remodeling (ADAMTS2), and cellular stress response (PARK2, RPTOR), supporting a mechanistic role for greenspace in modulating biological aging.

### 3.5. Enriched Molecular Pathways

To elucidate the biological mechanisms underlying greenspace-associated DNA methylation changes, four studies performed functional enrichment analyses of genes annotated to DMPs and DMRs. These analyses consistently identified pathways related to neurodevelopment, immune regulation, inflammation, and stress response.

In the SAPALDIA cohort, Jeong et al. reported that DMRs associated with proximal greenspace (30 m buffer) were enriched in pathways related to allergy, physical activity, and allostatic load, whereas distal greenspace (500 m buffer) was primarily associated with immune and stress-related pathways [10]. This suggests that different spatial scales of greenspace capture distinct exposure mechanisms, with proximal environments reflecting direct behavioral interactions and distal environments representing broader environmental quality.

In a multi-cohort analysis, Aguilar-Lacasaña et al. linked DMRs to genes involved in extracellular matrix remodeling (ADAMTS2), genomic imprinting and metabolism (KCNQ1DN), neurotransmitter transport (SLC6A12), and synaptic development (SDK1), highlighting potential roles in neurodevelopment and systemic regulation [19].

More comprehensive pathway analyses from the ENVIRONAGE cohort further identified enrichment in immune processes (e.g., HLA-DRB5), serotonergic signaling (e.g., HTR2A), hypothalamic–pituitary–adrenal (HPA) axis regulation (e.g., NR3C1, CRH), neurodevelopment (e.g., BDNF), and inflammatory pathways (e.g., IL6, TNF, NFKB1) [20]. The prominence of HPA axis and stress-related pathways is particularly relevant given the established role of stress reduction as a primary mechanism linking greenspace exposure to health outcomes.

Additional evidence supports the functional relevance of specific loci. For example, methylation of the HTR2A promoter was associated with greenspace exposure in placental tissue, suggesting modulation of serotonergic signaling during early development [18]. Similarly, methylation changes at PARK2, a gene implicated in neurodegeneration and tumor suppression, have been associated with adverse clinical outcomes, supporting the biological significance of genes identified in greenspace-related DMR analyses [31,63].

Finally, candidate gene analyses in children demonstrated enrichment in neurotransmitter-related pathways, including dopaminergic and glutamatergic signaling, which were associated with both greenspace exposure and cognitive outcomes (e.g., IQ), suggesting a potential role in neurodevelopmental processes [16].

Overall, these findings converge on three major biological themes: (1) neurodevelopment and synaptic plasticity (e.g., HTR2A, BDNF, SLC6A3, SDK1), (2) immune and inflammatory regulation (e.g., HLA family, IL6, TNF), and (3) stress response and HPA axis activity (e.g., NR3C1, CRH). These pathways provide a coherent mechanistic framework through which greenspace exposure may influence DNA methylation and contribute to the deceleration of biological aging.

### 3.6. Effect Modification by Genetic Background or Cell Type Composition

#### 3.6.1. Effect Modification by Genetic Background and Sociodemographic Factors

Two studies examined whether genetic background modifies the association between greenspace exposure and DNA methylation. Xu et al. (2021) reported significant interactions between greenness (NDVI/EVI) and single-nucleotide polymorphisms (SNPs) located within ±1 Mb of CpG sites, identifying 33 SNPs that modified CpG-specific associations (FDR < 0.05) [8]. These findings suggest that genetic variation may influence epigenetic responses to environmental exposures, particularly in genes related to neurodevelopment and immune function.

Similarly, Kim et al. (2023) reported effect modification by race, with higher NDVI associated with slower GrimAge acceleration in White participants (β = −3.03 years per IQR, 95% CI: −5.63 to −0.43), but not in Black participants (β = −0.80, 95% CI: −4.75 to 3.13) [1]. This heterogeneity persisted after adjustment for socioeconomic factors, suggesting that structural determinants (e.g., differential access to high-quality greenspace) and/or genetic ancestry may contribute to differential epigenetic responses.

In addition, Ward-Caviness et al. (2020) observed that neighborhood greenness modified the association between urban disadvantage and epigenetic mortality risk, further supporting the presence of context-dependent environmental effects [22,64].

#### 3.6.2. Sensitivity Analyses and Confounding Adjustment

All included studies adjusted for key confounders, including age, sex, smoking, and socioeconomic status, and most accounted for cellular heterogeneity using white blood cell composition or deconvolution methods [5,8,10,19,20,21]. Adjustment for air pollution (e.g., PM_2.5_, NO_2_) and proximity to major roads did not substantially alter the observed associations in several studies [19,20,22], suggesting that greenspace-related epigenetic effects are at least partially independent of traffic-related exposures.

However, results from the EXPANSE meta-analysis indicated that associations between NDVI and GrimAge acceleration in adults remained non-significant after pollution adjustment, whereas CpG-level associations in children were more robust [6]. This suggests potential differences in susceptibility across the life course.

#### 3.6.3. Consistency Across Studies and Potential Publication Bias

Despite heterogeneity in study design, populations, and exposure metrics, several patterns were consistently observed across studies. Greenspace exposure was associated with slower GrimAge acceleration [1,5,22], as well as with DMRs in genes such as KCNQ1DN [19,20] and with methylation changes in HTR2A [18,20]. In contrast, no consistent associations were observed for gestational epigenetic age acceleration in cord blood [21].

Associations with first-generation clocks (Horvath and Hannum) were less consistent, likely due to their lower sensitivity to environmental exposures [5,22]. Formal assessment of publication bias was not feasible due to the limited number of studies, although the presence of null findings reduces concerns regarding selective reporting.

### 3.7. Integrative Molecular Pathway Model

**Figure 2:**
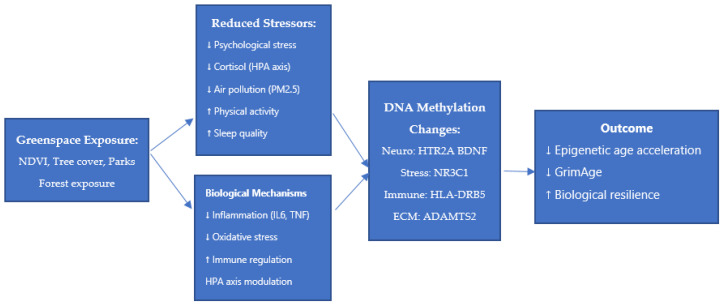
Molecular pathways linking greenspace exposure to epigenetic aging.

A schematic representation of the proposed molecular pathways linking greenspace exposure to epigenetic aging is presented in Figure 2.

Exposure to greenspace reduces environmental stressors, including psychological stress, cortisol levels, and air pollution, while promoting beneficial behaviors such as physical activity and improved sleep. These factors influence key biological mechanisms, including reduced inflammation, modulation of the hypothalamic–pituitary–adrenal (HPA) axis, and immune regulation. Consequently, these processes are associated with differential DNA methylation in genes involved in neurodevelopment (HTR2A, BDNF), stress response (NR3C1), immune regulation (HLA-DRB5), and extracellular matrix remodeling (ADAMTS2), ultimately contributing to the deceleration of epigenetic aging.

## 4. Discussion

### 4.1. Summary of Main Findings

This systematic review provides a comprehensive synthesis of evidence linking residential greenspace exposure to DNA methylation-based biomarkers of biological aging. Across 14 studies, the most consistent finding was that higher greenness (NDVI, tree cover, or vegetated land cover) is associated with deceleration of GrimAge, a second-generation epigenetic clock predictive of mortality and morbidity [1,5,22]. Effect sizes were modest but clinically meaningful, with reductions of approximately 1.0–1.6 years per interquartile range increase in greenness [5].

Associations with first-generation clocks (Horvath, Hannum) were less consistent, and third-generation clocks (e.g., DunedinPACE) remain underexplored. At the molecular level, greenspace exposure was associated with differential DNA methylation in genes involved in neurodevelopment (HTR2A, BDNF, SLC6A3), extracellular matrix remodeling (ADAMTS2), immune regulation (HLA-DRB5), and stress response (NR3C1, PARK2) [8,10,18,19,20,31].

Collectively, these findings support the concept of greenspace as an epigenomic resilience factor, capable of modulating key biological pathways that influence aging trajectories, potentially through coordinated effects on stress signaling, inflammation, oxidative balance, and epigenetic regulation.

### 4.2. Greenspace as an Epigenomic Resilience Factor: Proposed Molecular Mechanisms

The concept of epigenomic resilience refers to the capacity of environmental exposures to preserve or restore a favorable DNA methylation landscape, thereby slowing biological aging. Multiple, non-mutually exclusive mechanisms may explain how greenspace exerts these effects.

Stress reduction and HPA axis regulation: Chronic psychological stress accelerates epigenetic aging through glucocorticoid-mediated alterations in DNA methylation, particularly at genes such as NR3C1 and FKBP5 [32,33]. Greenspace exposure has been consistently associated with lower cortisol levels and reduced autonomic stress responses [34]. In this review, greenspace was associated with methylation changes in CRH and NR3C1 [20,34], supporting the hypothesis that reduced HPA axis activation may limit stress-induced epigenetic drift.

Immune and inflammatory modulation: Chronic low-grade inflammation (“inflammaging”) contributes to accelerated biological aging. Greenspace exposure has been associated with reduced levels of pro-inflammatory cytokines such as IL-6 and TNF-α [35]. Correspondingly, DMRs identified in this review were enriched in immune-related genes, including HLA-DRB5, IL6, and TNF [20,36,37]. Reduced exposure to pollutants in greener environments may further contribute to maintaining immune epigenetic homeostasis.

An additional indirect validation of our findings comes from comparing the observed greenspace-associated deceleration of epigenetic aging with the well-established acceleration of epigenetic aging in age-related inflammatory diseases. Chronic inflammatory conditions, including cardiovascular disease, neurodegenerative disorders, and metabolic syndromes, are consistently associated with accelerated epigenetic aging, reflecting the cumulative burden of systemic inflammation and oxidative stress [2,11].

In contrast, greenspace exposure, which is known to reduce circulating inflammatory markers and attenuate psychological stress, is associated with the opposite pattern: decelerated epigenetic aging. This inverse relationship supports the biological plausibility of greenspace as an epigenomic resilience factor [32]. Notably, several genes identified in our synthesis as differentially methylated in relation to greenspace (e.g., HLA-DRB5, IL6, TNF, NR3C1) are directly implicated in inflammatory and stress-response pathways, providing a mechanistic basis for this contrast. Thus, the epigenetic signatures associated with greenspace exposure are consistent with a shift away from the pro-inflammatory, accelerated-aging phenotype observed in chronic disease.

Oxidative stress and epigenetic regulation: Oxidative stress is a central driver of epigenetic aging, influencing both DNA damage and the activity of DNA methylation machinery. Reactive oxygen species (ROS) can alter the function of DNA methyltransferases (DNMTs), leading to aberrant methylation patterns and epigenetic drift. Greenspace exposure may mitigate oxidative stress through multiple pathways, including increased exposure to phytoncides, enhanced antioxidant capacity, and reduced environmental pollutants [38,39]. Supporting this mechanism, several studies identified enrichment of oxidative stress-related pathways among greenspace-associated DMRs [10,40], suggesting that reduced oxidative burden may help stabilize DNMT activity and preserve epigenomic integrity.

Epigenetic enzymatic regulation (DNMTs): Beyond indirect effects, environmental exposures are known to influence the expression and activity of epigenetic enzymes, particularly DNMT1, DNMT3A, and DNMT3B. Plant-derived bioactive compounds (e.g., flavonoids, terpenes) commonly present in natural environments have been shown to modulate DNMT activity and DNA methylation patterns [41,42]. Although direct human evidence remains limited, the consistent identification of coordinated methylation changes across multiple genomic regions supports the hypothesis that greenspace exposure may influence global epigenetic regulation through modulation of methyltransferase activity.

Physical activity and metabolic signaling: Physical activity, strongly promoted by greenspace, is a well-established modifier of DNA methylation, particularly in genes involved in energy metabolism and inflammation [43,44]. Enrichment of physical activity-related CpGs in proximity-based greenspace analyses supports this behavioral–epigenetic pathway [10].

Air pollution mitigation: Air pollution is a major driver of epigenetic age acceleration [36,45,46]. Greenspace reduces ambient exposure to PM_2.5_ and NO_2_, which are known to induce methylation changes in inflammatory and oxidative stress pathways. While adjustment for pollutants did not fully explain greenspace associations [19,20], evidence suggests that reduced pollutant exposure likely acts synergistically with other mechanisms.

Overall, these pathways converge on a central mechanism whereby greenspace exposure reduces systemic stress (psychological, inflammatory, and oxidative) and stabilizes epigenetic regulatory processes, ultimately contributing to slower epigenetic aging.

### 4.3. Comparison with Other Environmental Exposures

The magnitude of epigenetic age deceleration associated with greenspace exposure (1.0–1.6 years per interquartile range increase) is comparable to, or in some cases exceeds, that reported for other modifiable lifestyle factors. For example, healthy diet and regular physical activity have been associated with reductions of approximately 0.5–1.5 years in epigenetic age, while smoking cessation may reverse epigenetic aging by up to 1–2 years [47,48]. In contrast, adverse environmental exposures such as air pollution have been consistently associated with 1–3 years of age acceleration [36,45].

These comparisons highlight the potential clinical and public health relevance of greenspace as a modifiable environmental determinant of biological aging, particularly in urban settings where exposure disparities are pronounced.

### 4.4. Heterogeneity and Null Findings

Despite generally consistent trends, not all studies reported significant associations. Null findings were observed in studies examining gestational epigenetic age acceleration in cord blood [21,49] and in the EXPANSE meta-analysis of adult populations [6]. However, the absence of association with gestational epigenetic clocks in these specific studies should not be interpreted as evidence against the broader phenomenon of fetal epigenetic programming by environmental exposures.

Historical and contemporary evidence consistently demonstrates that adverse conditions during gestation, as well as beneficial exposures such as greenspace, can induce lasting epigenetic modifications that influence health trajectories well into adulthood [2,14]. These effects are well documented at the level of specific genes and are associated with long-term risks of cardiometabolic and psychiatric disorders [2]. Nevertheless, the null findings in the present context may reflect the limited sensitivity of gestational epigenetic clocks to detect greenspace-related effects, as these clocks were primarily trained to predict gestational age rather than to capture the cumulative impact of environmental exposures. Other epigenetic metrics, such as tissue-specific methylation at candidate genes, or third-generation clocks like DunedinPACE, may be more appropriate for capturing the subtle and beneficial effects of greenspace exposure during fetal development.

Several factors may explain this heterogeneity. First, developmental stage may influence susceptibility, as epigenetic clocks developed for gestational age are less sensitive to environmental exposures than adult-based clocks. Second, differences in clock selection may contribute, as first-generation or gestational clocks capture distinct biological processes. Third, exposure assessment methods, particularly the use of broad NDVI buffers, may not accurately reflect accessible or functionally relevant greenspace. Finally, population-level variability, including differences in urban design and greenspace quality, may contribute to inconsistent findings across cohorts.

### 4.5. Limitations of the Evidence Base

Several limitations should be considered. First, most included studies were cross-sectional, limiting causal inference. Second, substantial heterogeneity in greenspace metrics (e.g., NDVI, tree cover, proximity measures) complicates cross-study comparability and prevents identification of optimal exposure thresholds.

Third, residual confounding by socioeconomic status remains a concern, as individuals with higher socioeconomic status are more likely to reside in greener environments and exhibit healthier behaviors. Although most studies adjusted for individual and neighborhood-level confounders, bias cannot be fully excluded.

Fourth, tissue specificity may influence findings, as most studies relied on blood or cord blood, while greenspace-related epigenetic effects may differ across tissues such as brain or placenta. Finally, the small number of studies limited formal assessment of publication bias, although the inclusion of null findings reduces concerns regarding selective reporting.

### 4.6. Strengths

This review represents the first systematic synthesis of evidence linking greenspace exposure to DNA methylation-based biomarkers of biological aging. Strengths include adherence to PRISMA 2020 guidelines [29], the integration of observational and epigenome-wide association studies, and the identification of convergent molecular pathways.

The application of a structured PECO framework and the inclusion of studies reporting null findings enhance the robustness and transparency of the evidence synthesis.

### 4.7. Implications for Future Research

Future research should prioritize longitudinal designs with repeated measures of both greenspace exposure and DNA methylation to establish temporal relationships. The use of third-generation epigenetic clocks, such as DunedinPACE, may improve sensitivity to environmental influences [50,56].

Integration of multi-omics approaches (e.g., methylation, transcriptomics, metabolomics) will be critical to elucidate causal pathways. Studies should also examine life-course exposure windows, as epigenetic plasticity varies across developmental stages.

Standardization of greenspace metrics and the use of high-resolution spatial data would improve comparability across studies. Finally, investigation of effect modification by genetic ancestry, sex, and socioeconomic status, as well as intervention studies (e.g., greening programs), are needed to strengthen causal inference [51].

### 4.8. Public Health and Urban Planning Implications

Although this review focuses on molecular mechanisms, the findings have clear translational implications. Increasing access to greenspace, particularly tree cover and vegetated land, may contribute to population-level reductions in biological aging.

Given that epigenetic age acceleration is associated with increased risk of mortality, cancer, and neurodegenerative disease, even modest reductions (1–2 years) may have significant public health impact [52,53]. Importantly, observed disparities in greenspace benefits across populations highlight the need for equitable urban planning strategies that prioritize access in underserved communities.

From a mechanistic perspective, interventions that increase greenspace exposure may act through reduction in stress, inflammation, and oxidative damage, as well as through stabilization of epigenetic regulatory processes, reinforcing the role of the built environment as a determinant of molecular health.

## 5. Conclusions

This systematic review demonstrates that residential greenspace exposure is consistently associated with slower epigenetic aging, particularly as measured by second-generation epigenetic clocks such as GrimAge. The magnitude of this effect, approximately 1.0–1.6 years of age deceleration per interquartile range increase in greenness, is comparable to that of other key modifiable lifestyle and environmental factors.

At the molecular level, greenspace exposure is associated with differential DNA methylation in genes involved in neurodevelopment (e.g., HTR2A, BDNF, SLC6A3), immune and inflammatory regulation (e.g., HLA-DRB5, IL6, TNF), stress response and HPA axis activity (e.g., NR3C1), and extracellular matrix remodeling (e.g., ADAMTS2). These convergent pathways support a mechanistic framework in which greenspace contributes to the maintenance of epigenomic stability.

Emerging evidence suggests that greenspace may act as an epigenomic resilience factor, potentially through integrated mechanisms including reduction in psychological stress, attenuation of chronic inflammation, mitigation of oxidative stress, and stabilization of DNA methylation machinery, including DNA methyltransferase activity.

Despite heterogeneity across studies, the overall evidence indicates that greenspace is a biologically relevant environmental determinant of epigenetic aging. However, the predominance of cross-sectional designs, variability in exposure assessment, and potential residual confounding limit causal inference.

Future research should prioritize longitudinal and intervention-based designs, the use of next-generation epigenetic clocks, and integration of multi-omics approaches to clarify causal pathways. From a translational perspective, increasing equitable access to greenspace may represent a feasible population-level strategy to promote healthy aging.

Overall, these findings provide a molecular basis for the health benefits of greenspace and highlight the role of the built environment as a modifiable determinant of biological aging.

## Figures and Tables

**Table 1:** Risk of bias assessment of the 14 included studies.

Study (Year)	Tool	Selection	Comparability	Exposure/Outcome	Other Domains	Overall Risk
Kim et al. (2023) [1]	NOS	★★★	★★	★★★	--	Low (8/9)
Egorov et al. (2025) [5]	NOS	★★★	★★	★★★	--	Low (8/9)
Nazzari et al. (2026) [7]	NOS	★★★	★★	★★★	--	Low (8/9)
Xu et al. (2021) [8]	NOS	★★★	★★★	★★★	--	Low (9/9)
Zhao et al. (2025) [9]	NOS	★★★	★★	★★★	--	Low (8/9)
Jeong et al. (2022) [10]	ROBINS-I	Low	Low	Low	Moderate	Moderate
Vos et al. (2024) [15]	NOS	★★★	★★	★★★	--	Low (8/9)
Lee et al. (2021) [16]	NOS	★★★	★★	★★	--	Low (8/9)
De Ryck et al. (2024) [17]	NOS	★★★	★★	★★	--	Low (8/9)
Dockx et al. (2022) [18]	NOS	★★★	★★	★★	--	Low (8/9)
Aguilar-Lacasaña et al. (2024) [19]	ROBINS-I	Low	Low	Low	Low	Low
Alfano et al. (2023) [20]	ROBINS-I	Low	Low	Low	Low	Low
Marques et al. (2023) [21]	ROBINS-I	Low	Low	Low	Low	Low
Ward-Caviness et al. (2020) [22]	NOS	★★★	★★	★★	--	Low (8/9)

Nota. NOS (Newcastle–Ottawa Scale): maximum 9 stars (Comparability: 2 stars; Outcome/Exposure: 3 stars). Scores ≥7 = low risk of bias; 5–6 = moderate risk; ≤4 = high risk. ROBINS-I (Risk Of Bias In Non-randomized Studies of Interventions): domains include confounding, selection, classification of exposures, missing data, outcome measurement, and selective reporting. Overall judgment: Low, Moderate, Serious, Critical.

**Table 2:** Summary of included studies on greenspace exposure and DNA methylation-based epigenetic aging.

Study (Year)	Design	Population	Tissue	Greenspace Metric	Epigenetic Biomarker/Clock	Main Molecular Finding
Ward-Caviness et al. (2020) [22]	Cross-sectional	157 adults (Detroit)	Blood	Tree cover (500 m), abandoned cars, poor streets	eMRS (epigenetic mortality risk score)	Tree cover associated with lower eMRS (β = −0.59 years per IQR, 95% CI: 0.18–1.01); effect attenuated by large mature trees
Xu et al. (2021) [8]	Cross-sectional (twin-based)	479 Australian women	Blood	NDVI, EVI (300–2000 m)	EWAS (450K array)—CpG-specific	cg04720477 (CNP promoter) associated with NDVI (FDR < 0.05); 35 DMRs identified
Lee et al. (2021) [16]	Prospective cohort	59 children (age 2)	Blood	NDVI (GIS)	CpGs from cognitive-ability GWAS	25 CpGs significantly associated with greenness; cg26269038 (SLC6A3) associated with IQ at age 6
Jeong et al. (2022) [10]	Cross-sectional	982 SAPALDIA participants	Blood	NDVI (30 m, 500 m)	EWAS (EPIC array)—DMR analysis	163 DMRs for green30, 56 DMRs for green500; enrichment in allergy and allostatic load pathways
Dockx et al. (2022) [18]	Cross-sectional (ENVIRONAGE)	327 placentas	Placenta	Total green space (buffers 50 m–3 km), presence of nature	HTR2A promoter methylation	IQR increase in green space (1000 m) associated with 1.47% higher HTR2A methylation (95% CI: 0.17–2.78)
Kim et al. (2023) [1]	Prospective cohort	482 adults (CARDIA)	Blood (whole)	NDVI (5 km buffer)	GrimAge acceleration	Higher NDVI associated with slower GrimAge acceleration in white participants (β = −3.03 years per IQR); effect attenuated in Black participants
Alfano et al. (2023) [20]	Cross-sectional (ENVIRONAGE)	538 newborns	Cord blood	Green space (high/low vegetation, 100–1000 m)	EWAS (450K/EPIC)—DMR analysis	147 DMRs; 85 remained significant after PM2.5 adjustment; genes included HTR2A, RPTOR, KCNQ1DN
Marques et al. (2023) [21]	Cross-sectional (Generation R)	1359 newborns	Cord blood	NDVI, distance to green/blue space (300–3000 m)	Bohlin’s and Knight’s epigenetic gestational age clocks	No significant association with gestational age acceleration (*p* > 0.05)
Aguilar-Lacasaña et al. (2024) [19]	Multi-cohort cross-sectional	2988 newborns + 1849 children	Cord blood, child blood	NDVI (100, 300 m)	EWAS (450K/EPIC)—DMR analysis	4 DMRs annotated to ADAMTS2, KCNQ1DN, SLC6A12, SDK1 (FDR < 0.05)
Vos et al. (2024) [15]	Prospective cohort	52 mother-offspring pairs (follow-up 28–29 years)	Blood, saliva	Green space (high-resolution map)	NR3C1, IGF2/H19 methylation	Maternal stress associated with NR3C1 methylation; greenspace associated with IGF2/H19 methylation in saliva
De Ryck et al. (2024) [17]	Prospective cohort	120 mother-child pairs (conception to 5 years)	Buccal	Urban green, forest (high-resolution)	LEP methylation	Larger green clusters associated with lower LEP methylation (protective); smaller clusters opposite effect
Egorov et al. (2025) [5]	Cross-sectional	116 adults (North Carolina)	Blood	Tree cover, vegetated land cover, NDVI (500 m)	Horvath, Hannum, PhenoAge, GrimAge	IQR increase in tree cover → deceleration of 1.0–1.6 years across all four clocks (*p* < 0.05)
Zhao et al. (2025) [9]	Cross-sectional	156,690 UK Biobank participants	Blood	NDVI (1000 m buffer)	PhenoAge acceleration	NDVI negatively associated with PhenoAge acceleration (β = −0.003 per 0.1 NDVI, *p* = 2.28 × 10^−7^)
Nazzari et al. (2026) [7]	Prospective cohort	110 mother-infant dyads	Buccal (newborns)	Green space availability (CLCplus, 300 m)	BDNF methylation (11 CpGs)	Greenspace buffered effect of maternal anxiety on BDNF methylation (interaction *p* < 0.05)

Nota. NDVI = Normalized Difference Vegetation Index; EVI = Enhanced Vegetation Index; IQR = interquartile range; EWAS = epigenome-wide association study; DMR = differentially methylated region; FDR = false discovery rate; CI = confidence interval; CARDIA = Coronary Artery Risk Development in Young Adults; SAPALDIA = Swiss Cohort Study on Air Pollution and Lung and Heart Diseases in Adults; ENVIRONAGE = ENVIRonmental influence ON early aging; GIS = geographic information system; CLCplus = Corine Land Cover plus.

## Data Availability

No new data were created or analyzed in this study. Data sharing does not apply to this review article.

## Supplementary Materials

The following supporting information can be downloaded at: https://www.mdpi.com/article/10.3390/ijms27146538/s1. Another Figure used in this study is available at: https://drive.google.com/drive/folders/1B9Hm8srIDYvIry4_7576yWBULrhFsOLF?usp=sharing (accessed on 17 May 2026). Reference [24] is cited in the Supplementary Materials.

## Abbreviations

The following abbreviations are used in this manuscript:

BDNF	Brain-derived neurotrophic factor
BiSC	Barcelona Life Study Cohort
CARDIA	Coronary Artery Risk Development in Young Adults
CpG	Cytosine-phosphate-guanine dinucleotide
CRH	Corticotropin-releasing hormone
DMP	Differentially methylated position
DMR	Differentially methylated region
DNAm	DNA methylation
DNMT	DNA methyltransferase
ENVIRONAGE	ENVIRonmental influence ON early AGEing
eMRS	Epigenetic mortality risk score
EVI	Enhanced Vegetation Index
PRISMA	Preferred Reporting Items for Systematic Reviews and Meta-Analyses
EWAS	Epigenome-wide association study
EXPANSE	EXposome Powered by a Sustainability Approach
FDR	False discovery rate
GO	Gene Ontology
HPA	Hypothalamic–pituitary–adrenal
HTR2A	5-hydroxytryptamine receptor 2A
IL6	Interleukin 6
INMA	Infancia y Medio Ambiente (Environment and Childhood)
IQR	Interquartile range
KEGG	Kyoto Encyclopedia of Genes and Genomes
NDVI	Normalized Difference Vegetation Index
NOS	Newcastle–Ottawa Scale
NR3C1	Nuclear receptor subfamily 3 group C member 1 (glucocorticoid receptor)
OSF	Open Science Framework
PECO	Population, Exposure, Comparator, Outcome
ROBINS-I	Risk Of Bias In Non-randomized Studies of Interventions
ROS	Reactive oxygen species
SAPALDIA	Swiss Cohort Study on Air Pollution and Lung and Heart Diseases in Adults
SES	Socioeconomic status
TNF	Tumor necrosis factor
