# Supplementary material for: Greenspace Exposure and DNA Methylation Age Acceleration: A Systematic Review and Molecular Pathway Analysis
Source: Int J Mol Sci. 2026 Jul 22;27(14):6538. doi: 10.3390/ijms27146538 (PMC13409795; doi:10.3390/ijms27146538)
Supplement: Supplementary file 1 [file ijms-27-06538-s001.zip › ijms-4353830-supplementary.pdf]

PRISMA 2020 Checklist

Section and Topic	Item #	Checklist item	Location where item is reported
**TITLE**			
Title	1	Identify the report as a systematic review.	Title page: "Greenspace Exposure and DNA Methylation Age Acceleration: A Systematic Review and Molecular Pathway Analysis
**ABSTRACT**			
Abstract	2	See the PRISMA 2020 for Abstracts checklist.	Abstract (single paragraph); includes background, methods, results, conclusions
**INTRODUCTION**			
Rationale	3	Describe the rationale for the review in the context of existing knowledge.	Introduction, paragraphs 1–5 (pp. 2–4).
Objectives	4	Provide an explicit statement of the objective(s) or question(s) the review addresses.	Introduction, final paragraph: "Therefore, the aim of this systematic review is to critically evaluate and synthesize the available human evidence on the association between greenspace exposure and DNA methylation-based biomarkers of biological aging..."
**METHODS**			
Eligibility criteria	5	Specify the inclusion and exclusion criteria for the review and how studies were grouped for the syntheses.	Section 4.4 (Inclusion and Exclusion Criteria) and PECO framework

Section and Topic	Item #	Checklist item	Location where item is reported
			described therein
Information sources	6	Specify all databases, registers, websites, organisations, reference lists and other sources searched or consulted to identify studies. Specify the date when each source was last searched or consulted.	Section 4.2: Scopus and Web of Science Core Collection; search conducted from inception to May 2026.
Search strategy	7	Present the full search strategies for all databases, registers and websites, including any filters and limits used.	Section 4.3 provides full Boolean strings for Scopus and Web of Science.
Selection process	8	Specify the methods used to decide whether a study met the inclusion criteria of the review, including how many reviewers screened each record and each report retrieved, whether they worked independently, and if applicable, details of automation tools used in the process.	Section 4.4 and 4.5: two independent reviewers screened records using Rayyan; disagreements resolved by consensus or third reviewer.
Data collection process	9	Specify the methods used to collect data from reports, including how many reviewers collected data from each report, whether they worked independently, any processes for obtaining or confirming data from study investigators, and if applicable, details of automation tools used in the process.	Section 4.6: four reviewers extracted data independently using a standardized Excel template; disagreements resolved by consensus or consultation with a fifth reviewer.
Data items	10a	List and define all outcomes for which data were sought. Specify whether all results that were compatible with each outcome domain in each study were sought (e.g. for all measures, time points, analyses), and if not, the methods used to decide which results to collect.	Section 4.6: variables extracted include epigenetic biomarker (Horvath, Hannum, PhenoAge, GrimAge, DunedinPACE,

PRISMA 2020 Checklist

Section and Topic	Item #	Checklist item	Location where item is reported
			EWAS CpG/DMR), main molecular finding (effect size, CI, p-value), etc.
	10b	List and define all other variables for which data were sought (e.g. participant and intervention characteristics, funding sources). Describe any assumptions made about any missing or unclear information.	Section 4.6: also extracted first author, publication year, country, study design, population characteristics, sample size, tissue type, greenspace metric, covariates adjusted for, quality assessment score.
Study risk of bias assessment	11	Specify the methods used to assess risk of bias in the included studies, including details of the tool(s) used, how many reviewers assessed each study and whether they worked independently, and if applicable, details of automation tools used in the process.	Section 4.8 and 4.8.1: NOS for observational studies, ROBINS-I for EWAS; four independent reviewers; disagreements resolved by consensus.
Effect measures	12	Specify for each outcome the effect measure(s) (e.g. risk ratio, mean difference) used in the synthesis or presentation of results.	Section 4.8 and Results (2.2): effect measures include beta coefficients, mean differences (years), odds ratios, and p-values.
Synthesis methods	13a	Describe the processes used to decide which studies were eligible for each synthesis (e.g. tabulating the study intervention characteristics and comparing against the planned groups for each synthesis (item #5)).	Section 4.4 and 4.8: studies grouped by epigenetic clock type and level of analysis (DNAmAge

PRISMA 2020 Checklist

Section and Topic	Item #	Checklist item	Location where item is reported
			acceleration, DMPs, DMRs).
	13b	Describe any methods required to prepare the data for presentation or synthesis, such as handling of missing summary statistics, or data conversions.	Section 4.6: data extraction into standardized Excel template; no imputation for missing data.
	13c	Describe any methods used to tabulate or visually display results of individual studies and syntheses.	Section 4.9 and Results: Table 1, Table 2, Figures 1 and 2.
	13d	Describe any methods used to synthesize results and provide a rationale for the choice(s). If meta-analysis was performed, describe the model(s), method(s) to identify the presence and extent of statistical heterogeneity, and software package(s) used.	Section 4.8: narrative synthesis (meta-analysis not feasible due to heterogeneity); no meta-analysis performed.
	13e	Describe any methods used to explore possible causes of heterogeneity among study results (e.g. subgroup analysis, meta-regression).	Section 2.6.1 and 3.4: subgroup analyses by race, developmental stage, tissue type, and clock selection.
	13f	Describe any sensitivity analyses conducted to assess robustness of the synthesized results.	Section 2.6.2: sensitivity analyses adjusting for air pollution, distance to roads, and neighborhood SES.
Reporting bias assessment	14	Describe any methods used to assess risk of bias due to missing results in a synthesis (arising from reporting biases).	Section 2.6.3 and 4.8: not feasible due to small number of studies; null findings included to reduce concern.
Certainty	15	Describe any methods used to assess certainty (or confidence) in the body of evidence for an outcome.	Not formally

PRISMA 2020 Checklist

Section and Topic	Item #	Checklist item	Location where item is reported
assessment			assessed; limitations discussed in Section 3.5.
**RESULTS**			
Study selection	16a	Describe the results of the search and selection process, from the number of records identified in the search to the number of studies included in the review, ideally using a flow diagram.	Section 4.5 and Figure 2 (PRISMA flow diagram): 97 records identified, 13 duplicates removed, 84 screened, 70 excluded, 14 assessed, 14 included.
	16b	Cite studies that might appear to meet the inclusion criteria, but which were excluded, and explain why they were excluded.	Section 4.4 and 4.5: examples of excluded studies (e.g., mercury methylation, bacterial transformation, cancer genetics) are provided; specific citations not listed but categories are described.
Study characteristics	17	Cite each included study and present its characteristics.	Table 1 provides characteristics of all 14 included studies; each is cited by reference number in the table.
Risk of bias in studies	18	Present assessments of risk of bias for each included study.	Table 2 presents NOS and ROBINS-I ratings for each study.
Results of individual studies	19	For all outcomes, present, for each study: (a) summary statistics for each group (where appropriate) and (b) an effect estimate and its precision (e.g. confidence/credible interval), ideally using structured tables or plots.	Table 1 includes effect estimates and precision (e.g., β, 95% CI, p-values) for each study.

PRISMA 2020 Checklist

Section and Topic	Item #	Checklist item	Location where item is reported
Results of syntheses	20a	For each synthesis, briefly summarise the characteristics and risk of bias among contributing studies.	Section 2.2–2.5 summarise characteristics and bias; Table 2 provides risk of bias.
	20b	Present results of all statistical syntheses conducted. If meta-analysis was done, present for each the summary estimate and its precision (e.g. confidence/credible interval) and measures of statistical heterogeneity. If comparing groups, describe the direction of the effect.	No meta-analysis performed; narrative synthesis presented.
	20c	Present results of all investigations of possible causes of heterogeneity among study results.	Section 2.6.1 and 3.4 discuss heterogeneity by race, tissue, clock type.
	20d	Present results of all sensitivity analyses conducted to assess the robustness of the synthesized results.	Section 2.6.2: sensitivity analyses adjusting for air pollution and SES.
Reporting biases	21	Present assessments of risk of bias due to missing results (arising from reporting biases) for each synthesis assessed.	Section 2.6.3: not formally assessed; null findings included to mitigate concern.
Certainty of evidence	22	Present assessments of certainty (or confidence) in the body of evidence for each outcome assessed.	Not formally assessed; strengths and limitations discussed in Section 3.5 and 3.6.
**DISCUSSION**			
Discussion	23a	Provide a general interpretation of the results in the context of other evidence.	Section 3.1, 3.2, 3.3.
	23b	Discuss any limitations of the evidence included in the review.	Section 3.5.
	23c	Discuss any limitations of the review processes used.	Section 3.5 (also Section 4.2 acknowledges exclusion of other

Section and Topic	Item #	Checklist item	Location where item is reported
			databases).
	23d	Discuss implications of the results for practice, policy, and future research.	Section 3.7 and 3.8.
**OTHER INFORMATION**			
Registration and protocol	24a	Provide registration information for the review, including register name and registration number, or state that the review was not registered.	Section 4.10: "The protocol was registered in the Open Science Framework (OSF) prior to conducting the review [64]." (no registration number provided; OSF project link not specified).
	24b	Indicate where the review protocol can be accessed, or state that a protocol was not prepared.	Section 4.10: protocol available upon request or as supplementary materials; OSF link not publicly provided.
	24c	Describe and explain any amendments to information provided at registration or in the protocol.	No amendments reported.
Support	25	Describe sources of financial or non-financial support for the review, and the role of the funders or sponsors in the review.	Funding section: "This research received no external funding."
Competing interests	26	Declare any competing interests of review authors.	Conflicts of Interest section: "The authors declare no conflicts of interest."
Availability of data, code and other materials	27	Report which of the following are publicly available and where they can be found: template data collection forms; data extracted from included studies; data used for all analyses; analytic code; any other materials used in the review.	Section 4.9 and 4.10: "the complete dataset of the 84 initially retrieved records has been archived in an Open Science Framework (OSF)

PRISMA 2020 Checklist

Section and Topic	Item #	Checklist item	Location where item is reported
			repository [64]"; also available upon request. Supplementary materials link provided.

From: Page MJ, McKenzie JE, Bossuyt PM, Boutron I, Hoffmann TC, Mulrow CD, et al. The PRISMA 2020 statement: an updated guideline for reporting systematic reviews. BMJ 2021;372:n71. doi: 10.1136/bmj.n71. This work is licensed under CC BY 4.0. To view a copy of this license, visit https://creativecommons.org/licenses/by/4.0/
